# Epitranscriptional regulation of TGF-**β** pseudoreceptor BAMBI by m^6^A/YTHDF2 drives extrinsic radioresistance

**DOI:** 10.1172/JCI172919

**Published:** 2023-12-15

**Authors:** Liangliang Wang, Wei Si, Xianbin Yu, Andras Piffko, Xiaoyang Dou, Xingchen Ding, Jason Bugno, Kaiting Yang, Chuangyu Wen, Linda Zhang, Dapeng Chen, Xiaona Huang, Jiaai Wang, Ainhoa Arina, Sean Pitroda, Steven J. Chmura, Chuan He, Hua Laura Liang, Ralph Weichselbaum

**Affiliations:** 1 Department of Radiation and Cellular Oncology and; 2 Ludwig Center for Metastasis Research, University of Chicago, Chicago, Illinois, USA.; 3 State Key Laboratory of Animal Nutrition, Institute of Animal Sciences of Chinese Academy of Agricultural Sciences, Beijing, China.; 4 Department of Chemistry, Department of Biochemistry and Molecular Biology, and Institute for Biophysical Dynamics, University of Chicago, Chicago, Illinois, USA.; 5 Department of Neurosurgery, University Medical Center Hamburg-Eppendorf, Hamburg, Germany.; 6 Shandong Cancer Hospital and Institute, Shandong First Medical University and Shandong Academy of Medical Sciences, Jinan, China.; 7 The Committee on Clinical Pharmacology and Pharmacogenomics and; 8 Howard Hughes Medical Institute, University of Chicago, Chicago, Illinois, USA.

**Keywords:** Immunology, Oncology, Monocytes, Radiation therapy

## Abstract

Activation of TGF-β signaling serves as an extrinsic resistance mechanism that limits the potential for radiotherapy. Bone morphogenetic protein and activin membrane-bound inhibitor (BAMBI) antagonizes TGF-β signaling and is implicated in cancer progression. However, the molecular mechanisms of BAMBI regulation in immune cells and its impact on antitumor immunity after radiation have not been established. Here, we show that ionizing radiation (IR) specifically reduces BAMBI expression in immunosuppressive myeloid-derived suppressor cells (MDSCs) in both murine models and humans. Mechanistically, YTH N6-methyladenosine RNA-binding protein F2 (YTHDF2) directly binds and degrades *Bambi* transcripts in an *N*^6^-methyladenosine–dependent (m^6^A-dependent) manner, and this relies on NF-κB signaling. BAMBI suppresses the tumor-infiltrating capacity and suppression function of MDSCs via inhibiting TGF-β signaling. Adeno-associated viral delivery of *Bambi* (AAV-Bambi) to the tumor microenvironment boosts the antitumor effects of radiotherapy and radioimmunotherapy combinations. Intriguingly, combination of AAV-Bambi and IR not only improves local tumor control, but also suppresses distant metastasis, further supporting its clinical translation potential. Our findings uncover a surprising role of BAMBI in myeloid cells, unveiling a potential therapeutic strategy for overcoming extrinsic radioresistance.

## Graphical abstract

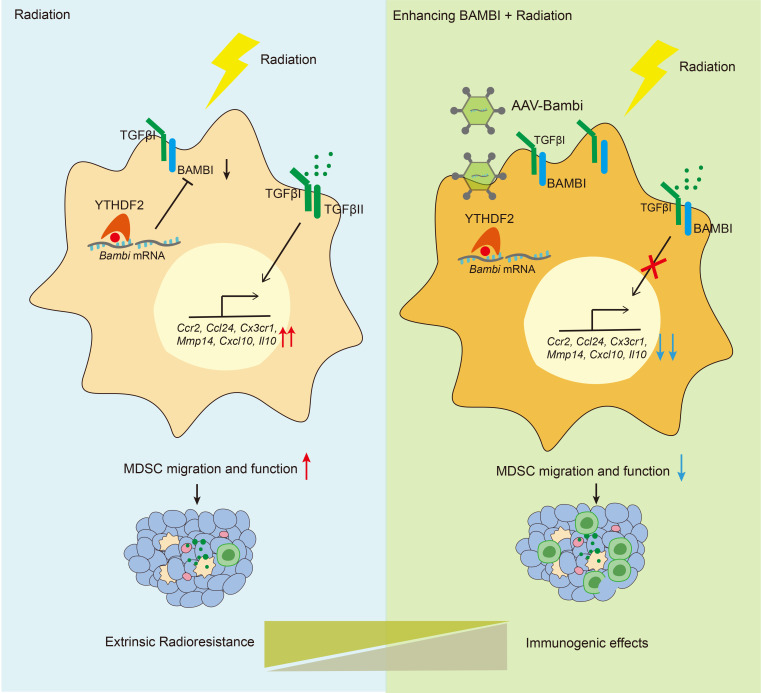

## Introduction

Radiation therapy (RT) is a common treatment option for cancer patients (1). Radiation simultaneously induces biological effects on cancer cells and elicits an immune response (2). However, the immunogenic effects of RT are counterbalanced by immune-suppressive effects and thereby contribute to treatment failure. Immune-suppressive mechanisms induced by RT include the following: recruitment of radioresistant suppressor cells (including myeloid-derived suppressor cells [MDSCs], Tregs, etc.) (3, 4) and induction of soluble suppressive factors, including programmed cell death ligand 1 (PD-L1) and TGF-β (5, 6). These can serve as factors of extrinsic radioresistance, highlighting the urgent need for alleviation of suppression in combination with RT to overcome extrinsic radioresistance for the benefit of patients.

MDSCs are immature myeloid-lineage cells that are specially associated with advanced cancer stage and adversely affect overall survival in cancer patients receiving RT (7–9). MDSCs suppress the antitumor immune response by secretion or expression of immunoregulatory factors (arginase 1 [ARG1], nitric oxide, TGF-β, IL-10, and others) (10, 11). Among these, targeting TGF-β has shown mixed results in the preclinical and clinical settings owing to its complex pleiotropic function as both oncogenic and tumor suppressive (12–14). Canonical TGF-β signaling is mostly attributed to the activation of SMAD2/3, whereas noncanonical signaling involves the activation of STAT1 (15). TGF-β signaling plays distinct roles in various immune cells. For example, loss of TGF-β signaling in T cells results in spontaneous development of gastrointestinal cancer (16). Additionally, TGF-β signaling can prevent the maturation of DCs (17). TGF-β induces chemotaxis in monocytes (18). Myeloid-specific TGF-β signaling acts as a critical mediator in tumor progression (19–21). Given that targeting TGF-β signaling in MDSCs may be an important therapeutic approach for enhancing RT efficacy, it is essential to clarify the regulation of TGF-β signaling in MDSCs.

Bone morphogenetic protein and activin membrane-bound inhibitor (BAMBI) is a transmembrane glycoprotein that has an extracellular ligand-binding domain structure similar to that of TGF-βR1, but lacks an intracellular serine/threonine kinase domain (22). BAMBI negatively regulates TGF-β signaling, acting as a TGF-β pseudoreceptor (23, 24). Previous studies have established that BAMBI is controlled by SMAD3/4 signaling and Wnt/β-catenin signaling in colon cancer cells (25, 26). DNA methylation is reported to be involved in BAMBI regulation in high-grade bladder cancer (27). These studies suggest that BAMBI expression is regulated at both the transcriptomic and epigenetic levels in cancer. Moreover, we recently showed that *N*^6^-methyladenosine (m^6^A) reader proteins and the epitranscriptome play a critical role in MDSC-suppressive function after ionizing radiation (IR) (28). However, the roles of epitranscriptional regulation and functional significance of BAMBI in immune cells are poorly understood.

Here, we report that IR reduces BAMBI expression in both human and mouse MDSCs through NF-κB–mediated induction of YTH N6-methyladenosine RNA-binding protein F2 (YTHDF2). YTHDF2 recognition of methylated *Bambi* transcripts signals them for degradation in an m^6^A-dependent manner. Importantly, BAMBI suppresses the tumor-infiltrating capacity and suppressive function of MDSCs by inhibiting TGF-β signaling. In murine models, either enhancing BAMBI expression in myeloid cells or in situ delivery of BAMBI via adeno-associated virus (AAV-Bambi) augmented the antitumor effects of radiotherapy and radioimmunotherapy combinations. Our findings uncover a mechanism by which BAMBI expression in myeloid cells can suppress pathways that cause extrinsic radioresistance and unveil a potential therapeutic strategy for cancer treatment.

## Results

### IR specifically reduces BAMBI expression in MDSCs in human and murine cancers.

We analyzed transcriptomic data sets of cancer patients to examine the potential relationship of BAMBI to cancer patient survival by leveraging the Kaplan-Meier plotter website (29). Higher BAMBI expression was associated with prolonged overall survival in 4 tumor types, including kidney renal clear cell carcinoma (*P* = 0.011), kidney renal papillary cell carcinoma (*P* = 0.045), pheochromocytoma and paraganglioma (*P* = 0.019), and uterine corpus endometrial carcinoma (*P* = 0.041) (Supplemental Figure 1A; supplemental material available online with this article; https://doi.org/10.1172/JCI172919DS1). Furthermore, our exploratory database analysis using TIMER2.0 (30) revealed that BAMBI was positively correlated with CD8^+^ T cell tumor infiltration and negatively correlated with MDSC tumor infiltration in both bladder carcinoma (BLCA) and KIRC (Supplemental Figure 1, B and C). Together, our in silico observations suggest that elevated BAMBI levels correlate with better survival and antitumor immunity among cancer patients.

**Figure 1:**
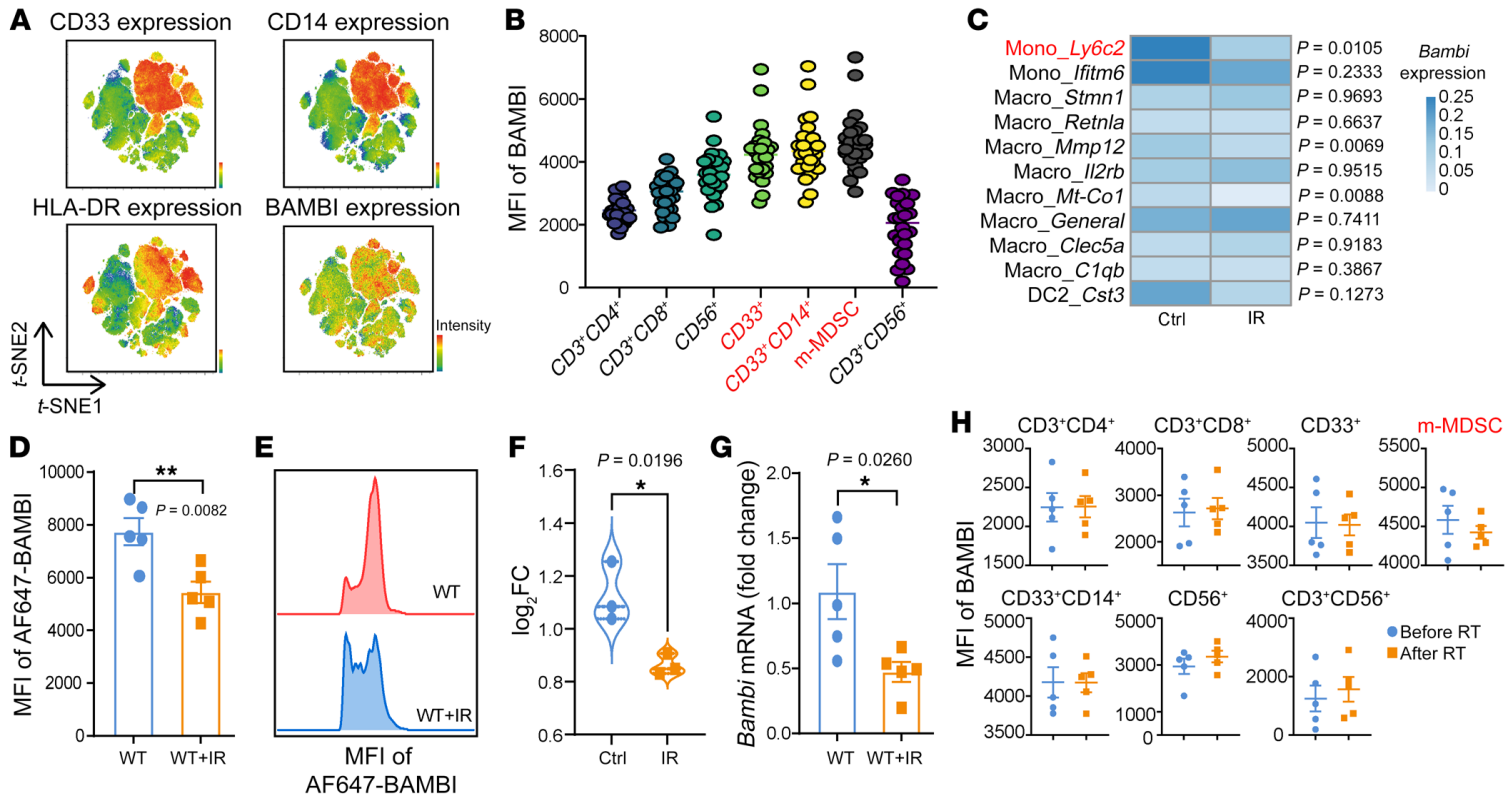
IR specifically reduces BAMBI expression in MDSCs in human and murine cancers. (**A**) *t*-SNE clustering of flow cytometry marker expression profiles in live CD45^+^ cells of PBMCs from metastatic NSCLC patients enrolled in a clinical trial (NCT03223155). Expression intensity of CD33, CD14, HLA-DR, and BAMBI. (**B**) MFI of BAMBI in different immune cells in PBMCs from metastatic NSCLC patients enrolled in a clinical trial (NCT03223155). We show the markers (CD33^+^CD14^hi^HLA-DR^lo^) used to gate m-MDSC. (**C**) Heatmap showing the changes of *Bambi* mRNA expression in different myeloid cell clusters in control (Ctrl) and IR tumors, respectively, based on the scRNA-Seq data of CD45^+^ cells. CD45^+^ cells were obtained from 4 pooled MC38 tumors 4 days after IR. *P* values were calculated by a Wilcoxon-Mann-Whitney test. (**D** and **E**) MFI of BAMBI (**D**) and representative flow cytometry analysis of BAMBI expression (**E**) in MC38 tumor–infiltrating MDSCs 3 days after IR. *n* = 5 per group for **D**. (**F**) TmRNA levels (log_2_ fold change-log_2_FC) of Bambi based on RNA-Seq data of CD11b^+^ myeloid cells isolated from nonirradiated or irradiated MC38 tumors 3 days after IR. (**G**) qPCR analysis of *Bambi* in MDSCs (CD45^+^CD11b^+^Ly6C^hi^) isolated from nonirradiated or irradiated MC38 tumors, 3 days after IR (20 Gy). *n* = 5 per group. (**H**) Flow cytometry analysis of BAMBI expression (MFI of BAMBI) in different immune cells in PBMCs from 5 metastatic NSCLC patients (enrolled in clinical trial NCT03223155) (pre-RT versus post-RT). The blood samples were collected immediately after hypofractionated radiation, approximately 1 to 3 weeks (median 14 days) after the pre-RT samples. Data are represented as means ± SEM. One of 2 or 3 representative experiments is shown (**D**, **E**, and **G**). Statistical analysis was performed using 2-sided, unpaired Student’s *t* test (**D**, **E**, and **G**). *P < 0.05; **P < 0.01.

Next, we used 2 murine cancer models to exclude the role of tumor cell–intrinsic BAMBI in antitumor activity. Overexpression or knockdown (KD) of BAMBI did not affect the in vivo tumor growth of either MC38 colon cancer or B16 melanoma (Supplemental Figure 1, D–I). We speculated that BAMBI may mainly function in immune cells. To verify this, we measured the expression levels of BAMBI in different immune cells in MC38 tumors by flow cytometry analysis and found that BAMBI is predominantly expressed on myeloid-associated cells, including monocytes, macrophages, and DCs (Supplemental Figure 2A). Such a scenario is consistent with our analysis using public single-cell RNA-Seq (scRNA-Seq) data in mice with colorectal adenocarcinoma (Supplemental Figure 2B). To dissect the main source of BAMBI in humans, we analyzed public scRNA-Seq data from the Tumor Immune Single-cell Hub 2 (TISCH2) (http://tisch.comp-genomics.org/) and observed the relatively high expression of BAMBI in monocytes/macrophages in patients with melanoma or colorectal cancer (Supplemental Figure 2C). Furthermore, we measured the protein levels of BAMBI in PBMCs from cancer patients enrolled in a clinical trial at our institution (Concurrent or sequential immunotherapy and radiation therapy in patients with metastatic lung cancer [COSINR] study; ClinicalTrials.gov NCT03223155) (31) and found monocytic MDSCs (mMDSCs) made up a sizable portion of BAMBI-positive cells (Figure 1, A and B, and Supplemental Figure 2D).

Considering the dominant role of myeloid cells in radioresistance (4, 28, 32), we sought to investigate the effect of IR on BAMBI expression in myeloid cell compartments. Our scRNA-Seq data on CD45^+^ immune cells isolated from irradiated (4 days after 20 Gy-IR) and nonirradiated MC38 tumors revealed that the mRNA level of *Bambi* was significantly decreased in the monocyte_*Ly6c2* population after IR (*P* = 0.0105; Figure 1C). We also observed a significant reduction in *Bambi* in the macrophage_*Mt-Co1* subset (*P* = 0.0088; Figure 1C). It is worth mentioning that monocyte_*Ly6c2*, identified as an mMDSC subset, exhibited the greatest proportionate increase among cell populations after IR, while a 2-fold decrease of macrophage_*Mt-Co1* populations was observed following IR, as mentioned in our previous study (28). We confirmed these findings by flow cytometry, demonstrating that BAMBI decreased in tumor-infiltrating mMDSCs (Figure 1, D and E), but remained unchanged in other immune cells or CD45^–^ cells, including tumor cells (Supplemental Figure 3, A and B). In addition, our bulk RNA-Seq and quantitative PCR (qPCR) analysis confirmed the decreased mRNA expression of *Bambi* following IR (Figure 1, F and G). In an effort to translate this finding to humans, we calculated the mRNA levels of BAMBI in biopsies from cancer patients (NCT03223155) (31); patients received stereotactic body radiotherapy (SBRT) and ipilimumab plus nivolumab (ipi/nivo) immunotherapy. BAMBI was modestly decreased after RT versus before RT in 6 out of 9 patients with lung or liver metastasis (Supplemental Figure 3C). We also evaluated the levels of BAMBI in PBMCs and observed a mild decrease of BAMBI in mMDSCs, not in other immune cells, from PBMCs following RT compared with matched pre-RT levels (Figure 1H). Our findings demonstrate that IR specifically reduces BAMBI expression in MDSCs in both human and murine cancers.

### IR regulates BAMBI expression in an m^6^A-YTHDF2–dependent manner.

**Figure 2:**
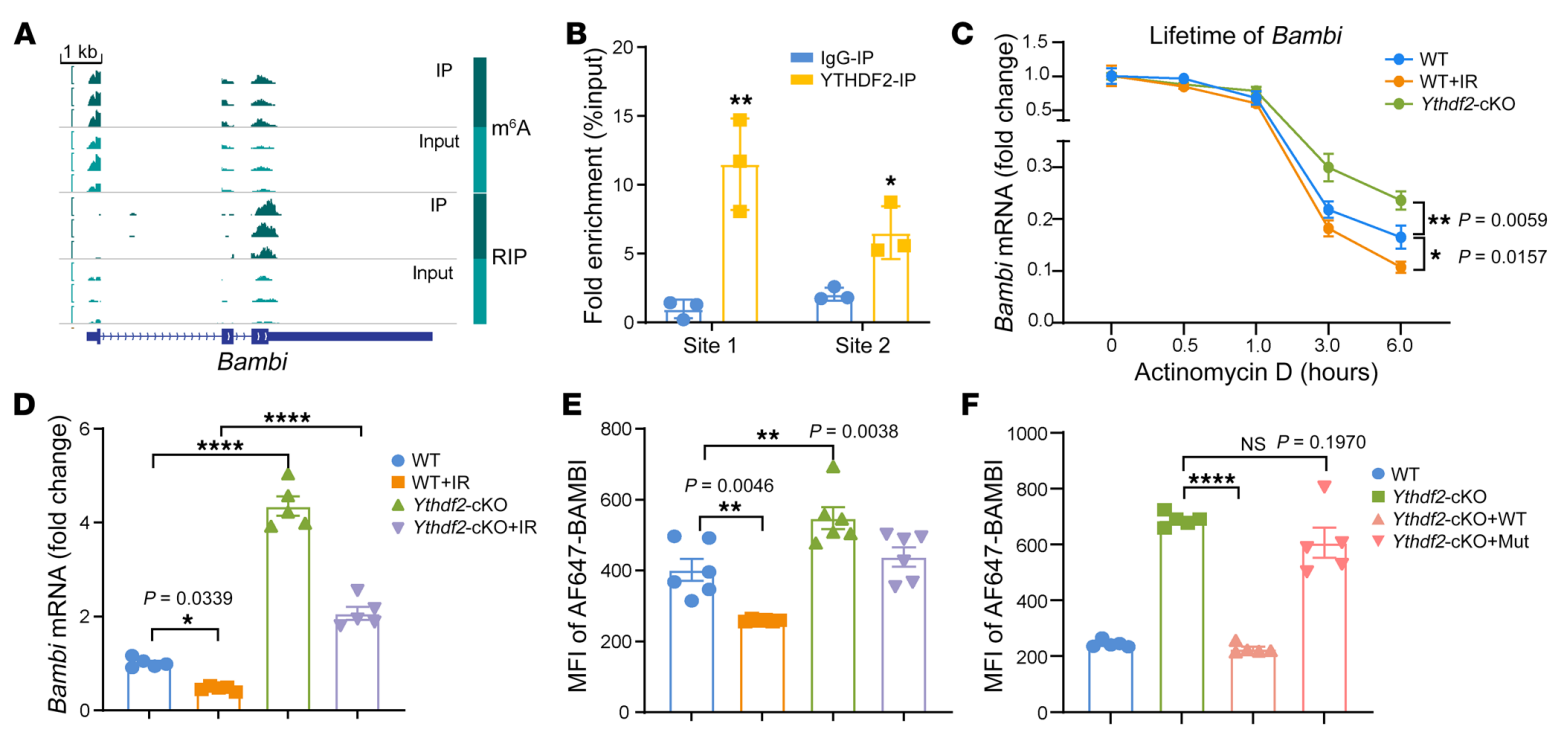
IR/YTHDF2 axis regulates BAMBI expression in MDSCs in an m^6^A-dependent manner. (**A**) Integrative Genomics Viewer tracks displaying the distribution of m^6^A peaks and YTHDF2-binding peaks across *Bambi* transcripts, based on the MeRIP-Seq and RIP-Seq of MC38 tumor–infiltrating myeloid cells. (**B**) Graphs showing enrichment of *Bambi* mRNA in the YTHDF2-immunoprecipitated RNA fraction of BM-MDSCs, determined by RIP-qPCR. Rabbit IgG served as a control. Enrichment of the indicated genes was normalized to the input level. *n* = 3 per group. (**C**) MDSCs were sorted from BM-derived cells from WT and *Ythdf2*-cKO (*Lyz2^cre^Ythdf2^fl/fl^*) mice; WT MDSCs were directly treated with IR (4 Gy and cultured for 6 hours) (WT+IR). MDSCs were treated with actinomycin D. mRNA was collected at indicated time points after treatment and mRNA levels of *Bambi* were measured by RT-qPCR. *n* = 3 per group. (**D**) qPCR analysis of *Bambi* mRNA levels in different MDSCs isolated from MC38 tumors in WT, WT+IR, *Ythdf2*-cKO, and *Ythdf2*-cKO+IR mice 3 days after IR. *n* = 5 per group. (**E**) MFI of BAMBI in MC38 tumor–infiltrating MDSCs (as indicated) by flow cytometry analysis 3 days after IR. *n* = 6 per group. (**F**) WT-YTHDF2 and m^6^A-binding site-mutated YTHDF2-overexpressing *Ythdf2*-deficient CD45.2-BM-MDSCs (*Ythdf2*-cKO+WT, *Ythdf2*-cKO+Mut, respectively) were obtained via lentiviral transfection. The BM-MDSCs were used for adoptive transfer into MC38 tumor–bearing CD45.1 mice. On the same day, mice were treated with local tumor irradiation. Three days after IR, tumors were harvested to measure the MFI of BAMBI in newly infiltrated CD45.2-MDSCs by flow cytometry. *n* = 5 per group. Data are represented as means ± SEM. One of 2 or 3 representative experiments is shown (**B**–**F**). Statistical analysis was performed using 2-sided, unpaired Student’s *t* test (**B**) or 1-way ANOVA with Bonferroni’s multiple-comparison tests (**C**–**F**). *P < 0.05; **P < 0.01; ****P < 0.0001.

We sought to ascertain the potential molecular mechanisms involved in radiation reduction of BAMBI in MDSCs. Considering that YTHDF2, an m^6^A reader protein, was dramatically induced by IR in myeloid cells (28), we reanalyzed a prior data set of *N*^6^-methyladenosine–sequencing RNA immunoprecipitation followed by high-throughput sequencing (MeRIP-Seq) and RNA immunoprecipitation sequencing (RIP-Seq) data of tumor-infiltrating CD11b^+^ myeloid cells to map direct target transcripts bound by YTHDF2. The results were highly reproducible among 3 biological replicates. The Integrative Genomics Viewer (https://igv.org/) indicated a good fit between the m^6^A peaks and the YTHDF2-binding peaks in the 3′ UTR of *Bambi* (Figure 2A). RIP-qPCR analyses with anti-YTHDF2 indicated that 2 sites were enriched in YTHDF2 binding in MDSCs compared with that with IgG control (Figure 2B). Moreover, the transcript half-time of *Bambi* mRNA was significantly decreased in WT+IR MDSCs and increased in *Ythdf2*-cKO MDSCs (generated from *Lyz2*^cre^*Ythdf2^fl/fl^* conditional KO (cKO) mice) compared with WT MDSCs (Figure 2C), suggesting that IR promotes *Bambi* mRNA degradation via YTHDF2. We further confirmed the BAMBI reduction in tumor-infiltrating MDSCs from WT+IR versus WT mice and the BAMBI induction in MDSCs from *Ythdf2*-cKO versus WT mice at both mRNA and protein levels (Figure 2, D and E). Since IR can induce YTHDF2 via NF-κB/RELA signaling in MDSCs (28), we tested to determine whether RELA is involved in BAMBI regulation. We measured BAMBI expression in tumor-infiltrating MDSCs from *Lyz2*^cre^*Rela^fl/fl^* mice and found *Rela* KO can increase BAMBI expression and rescue the reduction of BAMBI (Supplemental Figure 4, A and B). These results highlight an IR/RELA/YTHDF2 axis that downregulates BAMBI in MDSCs after IR.

To further demonstrate whether the aforementioned phenotype requires the m^6^A-binding capacity of YTHDF2, we force expressed YTHDF2 (Ythdf2-WT) and m^6^A binding site–mutated YTHDF2 (Ythdf2-Mut) in *Ythdf2*-deficient BM-derived MDSCs (BM-MDSCs) (CD45.2) and adoptively transferred these cells into MC38 tumor–bearing CD45.1 mice, followed by IR treatment (20 Gy, 1 dose). Three days after IR, the BAMBI levels in newly infiltrated CD45.2-MDSCs in tumors were analyzed. Compared with the transferred WT MDSCs, *Ythdf2*-cKO MDSCs elicited significantly higher BAMBI levels (Figure 2F and Supplemental Figure 4C). Overexpression of YTHDF2-WT in *Ythdf2*-cKO MDSCs downregulated BAMBI expression to the levels in WT MDSCs, whereas YTHDF2-Mut overexpression did not change BAMBI expression levels, which were maintained at a similar level in *Ythdf2*-cKO MDSCs (Figure 2F and Supplemental Figure 4C). Furthermore, we performed m^6^A–RIP-qPCR analysis using BM-MDSCs and found that the BAMBI transcript was indeed methylated (Supplemental Figure 4D). Taken together, these observations indicate that an IR/RELA/YTHDF2 axis limits BAMBI production in MDSCs in an m^6^A-dependent manner.

### BAMBI suppresses MDSC migration and suppressive function via inhibiting TGF-β signaling.

**Figure 3:**
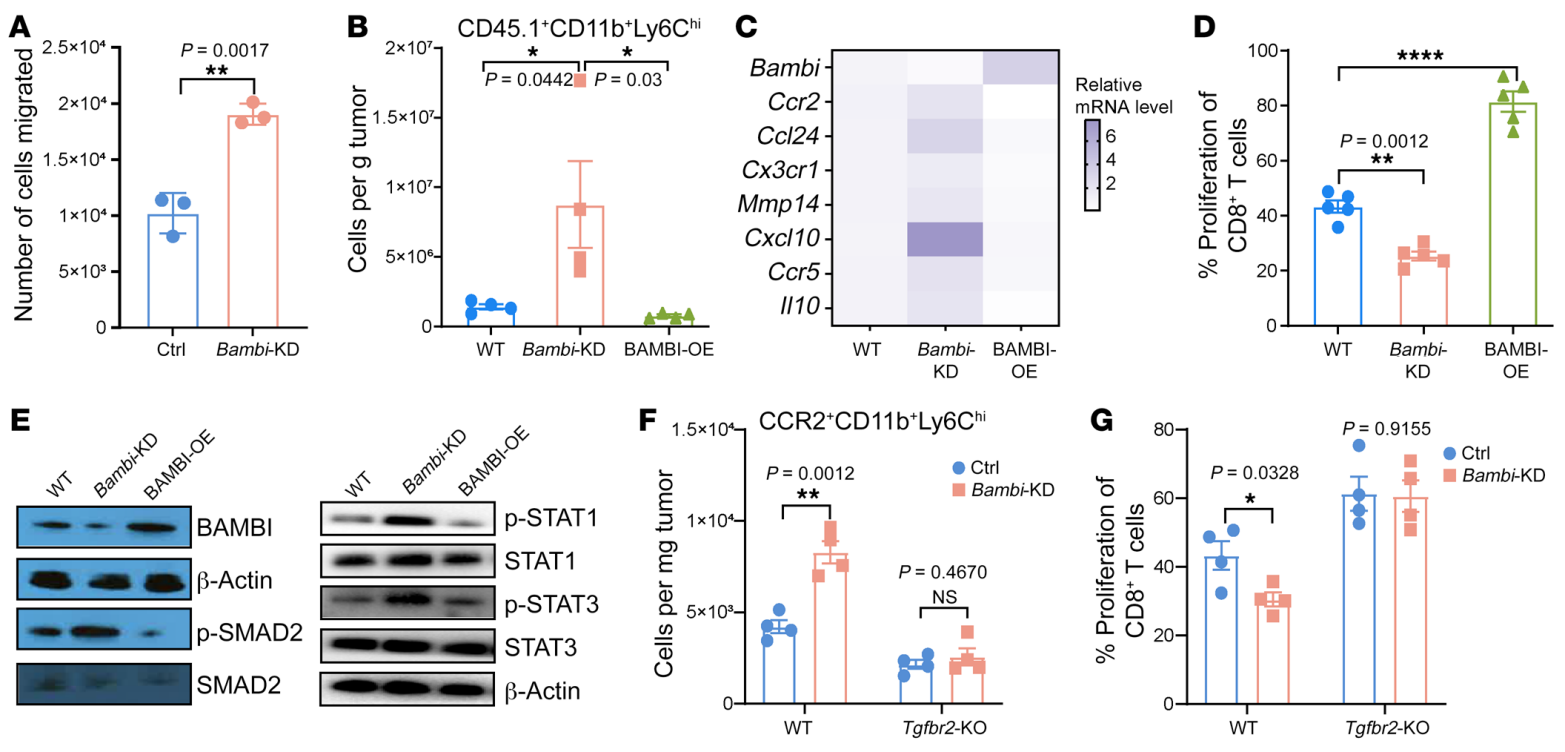
BAMBI suppresses MDSC migration and suppressive function via inhibiting TGF-β signaling. (**A**) WT and *Bambi*-KD BM-MDSCs as indicated were used for Transwell assay. The attached cells on the Transwell membrane were visualized under a light microscope and quantified. *n* = 3 per group. (**B**) MC38 tumor–bearing *Ccr2*-KO mice (CD45.2) were adoptively transferred with 1 × 10^6^ WT, *Bambi*-KD, and BAMBI-overexpressing (BAMBI-OE) BM-MDSCs (CD45.1) via i.v. injection. On the same day, mice were treated with tumor-local IR (20 Gy, 1 dose). Three days after IR, the number of tumor-infiltrating CD45.1^+^CD11b^+^Ly6C^hi^ cells was determined by flow cytometry. *n* = 4 per group. (**C**) Heatmap showing qPCR analysis of relative mRNA expression of indicated genes in WT, *Bambi*-KD, and BAMBI-OE MDSCs. qPCR data were normalized to *Gapdh*. *n* = 3 per group. (**D**) Flow cytometry analysis of an in vitro proliferation assay showing the frequency of proliferating CD8^+^ T cells when cocultured with WT, *Bambi*-KD, and BAMBI-OE MDSCs. *n* = 5 per group. (**E**) Immunoblot analysis of signaling associated proteins and phosphorylated (p-) proteins as indicated in WT, *Bambi*-KD, and BAMBI-OE MDSCs treated with murine recombinant TGF-β. Blots were run in parallel, and left and right panels were run at different times. (**F**) WT, *Bambi*-KD, *Tgfbr2*-KO, and *Bambi*-KD/*Tgfbr2*-KO BM-MDSCs were used for adoptive transfer into MC38 tumor–bearing *Ccr2*-KO mice. On the same day, mice were treated by with tumor-local IR. Three days after IR, the number of tumor-infiltrating CCR2^+^CD11b^+^Ly6C^hi^ cells was determined by flow. *n* = 4 per group. (**G**) Flow cytometry analysis of an in vitro proliferation assay showing the frequency of proliferating CD8^+^ T cells when cocultured with different BM-MDSCs as indicated. *n* = 4 per group. Data are represented as means ± SEM. One of 2 or 3 representative experiments is shown. Statistical analysis was performed using 2-sided, unpaired Student’s *t* test (**A**, **F**, and **G**) or 1-way ANOVA with Bonferroni’s multiple-comparison tests (**B** and **D**). *P < 0.05; **P < 0.01; ****P < 0.0001.

To investigate the functional role of BAMBI in MDSCs, we generated *Bambi*-KD BM-MDSCs using siRNA and measured their migration activity. Transwell assay showed that *Bambi*-KD MDSCs elicited significantly higher migration capacities than WT MDSCs (Figure 3A, *P* = 0.0017). Subsequently, we adoptively transferred *Bambi*-KD or BAMBI-overexpressing MDSCs (CD45.1) into MC38 tumor–bearing *Ccr2*-KO mice (CD45.2), in which radiation-induced MDSC infiltration is absent (4). Flow cytometry analysis revealed a significant increase of infiltrated MDSCs (CD45.1^+^CD11b^+^Ly6C^hi^) in mice transferred with *Bambi*-KD MDSCs, while a similar increase was not detected in mice transferred with BAMBI-overexpressing MDSCs upon radiation treatment (Figure 3B). To examine any changes in underlying migration-associated genes, we performed a series of qPCR analyses and found that the mRNA levels of *Ccr2*, *Ccl24*, *Cx3cr1, Ccr5, Cxcl10*, and *Mmp14* were markedly increased in *Bambi*-KD MDSCs and decreased in BAMBI-overexpressing MDSCs (Figure 3C). In addition, these genes were transcriptionally regulated by the YTHDF2/BAMBI axis, as evidenced by rescued mRNA expression in *Bambi*-KD/*Ythdf2*-cKO MDSCs compared with those in *Ythdf2*-cKO MDSCs (Supplemental Figure 5A). These results demonstrate that the YTHDF2/BAMBI axis regulates MDSC migration both in vitro and in vivo.

It is noteworthy that we observed that *Bambi*-KD led to an increase in *Il10*, whereas the overexpression of BAMBI decreased *Il10* at the mRNA level (Figure 3C). Importantly, this was not observed for other immune suppression mediators (*iNos2*, *Ros*, *Arg1*, and *Tgfb1*), suggesting that BAMBI was not involved in their regulation. To further confirm the role of BAMBI in MDSC-suppressive function, we cocultured *Bambi*-KD MDSCs with naive CD8^+^ T cells and detected a reduction in T cell proliferation (Figure 3D). The opposite result was observed when coculturing BAMBI-overexpressing MDSCs and CD8^+^ T cells (Figure 3D). Collectively, our results demonstrate that IR decreases BAMBI to enhance MDSC migration and suppressive function, thereby contributing to radioresistance.

It is well known that TGF-β signaling facilitates MDSC migration, suppressive function, and the related chemokine/cytokine regulation (19, 21). It is thus conceivable that the functional role of BAMBI in MDSCs requires TGF-β signaling. In pursuing this concept, we first sought to verify whether BAMBI impairs TGF-β signaling in MDSCs. We performed Western blots to detect the levels of phosphorylated SMAD2, a central downstream factor of TGF-β signaling, in WT or *Bambi*-KD MDSCs cocultured with recombinant TGF-β. SMAD2 phosphorylation was increased in *Bambi*-KD MDSCs and decreased in BAMBI-overexpressing MDSCs compared with WT MDSCs (Figure 3E). TGF-β can directly activate the JAK1/STAT1/3 axis in a non-SMAD manner (33, 34). In concert with this, our Western blot assay revealed the consistent changes in both STAT1 and STAT3 phosphorylation in *Bambi*-KD MDSCs and BAMBI-overexpressing MDSCs (Figure 3E). These findings indicate that BAMBI suppresses TGF-β signaling in MDSCs.

To further confirm the involvement of TGF-β signaling in *Bambi*-KD–induced MDSC infiltration, we generated *Bambi*-KD/*Tgfbr2*-KO MDSCs using BM cells (BMCs) from *Lyz^Cre+^Tgfbr2^fl/fl^* cKO mice (hereafter *Tgfbr2*-cKO) and used these constructs for the aforementioned transfer experiment. As expected, 3 days after IR, the levels of infiltrating MDSCs (CCR2^+^CD11b^+^Ly6C^hi^) were restored to levels similar to those of *Tgfbr2*-KO MDSC transfer (*P* = 0.4670; Figure 3F). *Bambi*-KD/*Tgfbr2*-KO MDSCs also exhibited suppressive function comparable to that of *Tgfbr2*-KO MDSCs (Figure 3G). Functionally, we observed consistent changes in the expression of genes associated with migration and suppressive functions of MDSCs, including *Ccr2*, *Ccl24*, *Cx3cr1*, *Mmp14*, *Cxcl10*, and *Il10* (Supplemental Figure 5B). To determine whether TGF-β in myeloid cells alters the response to radiotherapy, we employed *Tgfbr2*-cKO and *Tgfbr2^fl/fl^* (hereafter WT) for tumor-growth experiments. In the syngeneic murine colon carcinoma (MC38) model, although primary tumor growth in *Tgfbr2*-cKO mice was slower than that in WT mice, local IR resulted in a more pronounced inhibition of tumor growth in *Tgfbr2*-cKO mice (Supplemental Figure 5C). Taken together, these data demonstrate that IR downregulates BAMBI and the decreased BAMBI induces TGF-β–mediated MDSC immune suppression.

### Enhancing BAMBI expression improves the response to radiotherapy.

**Figure 4:**
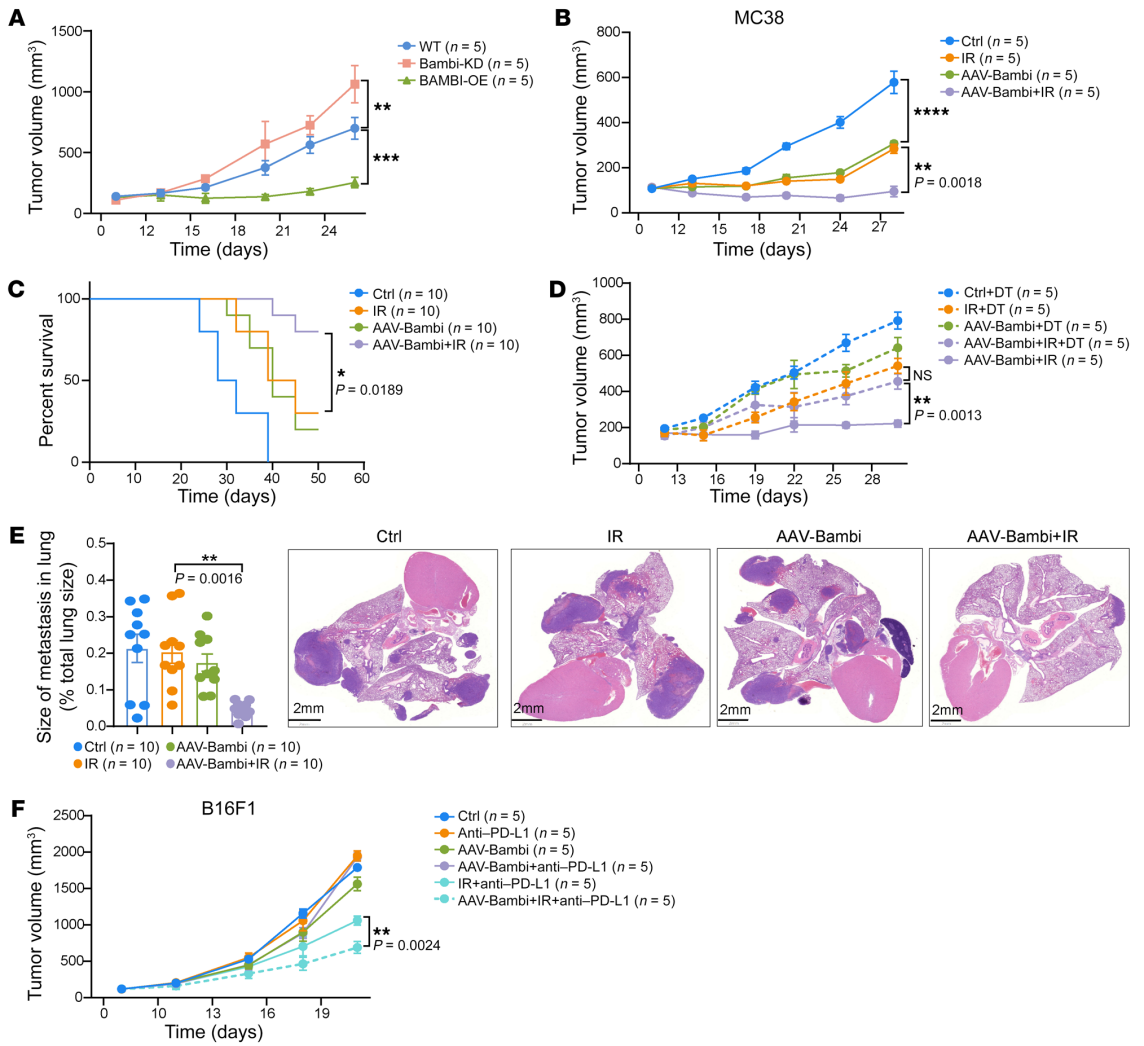
Enhancing BAMBI improves response to radiotherapy and immunotherapy. (**A**) CD11b-DTR BM chimeric mice were injected s.c. with 1 × 10^6^ MC38 cells. When the tumor size reached 100 mm^3^, mice were injected with DT (50 ng/per mouse). The next day, the mice were adoptive transferred with BM-MDSCs (CD45.1) as indicated and were treated with tumor-local IR (20 Gy, 1 dose). Tumor growth was monitored. *n* = 5 mice per group. (**B** and **C**) MC38 tumor–bearing mice were treated with tumor-local IR. On the same day, the mice were i.t. injected with 2 × 10^10^ v.p. of AAV-Null (Ctrl) or AAV-Bambi (twice weekly, 3 doses total). Tumor growth (**B**, *n* = 5 mice per group) and animal survival (**C**, *n* = 10 mice per group) were monitored. (**D**) *Lyz2*^cre^CSF1R-DTR mice were injected s.c. with 1 × 10^6^ MC38 cells. When the tumor size reached 100 mm^3^, mice were injected with DT (50 ng/per mice). The next day, mice were treated with tumor-local IR or AAV-Bambi (i.t. of 2 × 10^10^ v.p.). Tumor growth was monitored. *n* = 5 mice per group. (**E**) Lung metastasis in LLC tumor–bearing WT mice with indicated different treatments (22 days after IR). Size of lung metastases was measured. *n* = 10 mice per group. (**F**) B16F1 tumor–bearing WT mice were treated with anti–PD-L1 antibody (2 doses per week, 3 doses total), AAV-Bambi (twice weekly, 3 doses totally), and/or tumor-local IR, as indicated. Tumor growth was monitored. *n* = 5 mice per group. Data are represented as means ± SEM. Statistical analysis was performed using 2-way ANOVA with corrections for multiple variables (**A**, **B**, **D**, and **F**), 2-sided log-rank (Mantel-Cox) test (**C**), or 1-way ANOVA with Bonferroni’s multiple-comparison tests (**E**). *P < 0.05; **P < 0.01; ***P < 0.001; ****P < 0.0001.

To determine the role of myeloid-specific BAMBI in controlling tumor growth, we employed the BM chimeric mice with CD45.1 BMCs from CD11b-DTR mice for the following adaptive transfer experiments. Upon administration of diphtheria toxin (DT), WT myeloid cells expressing CD11b-DTR were selectively eliminated, leaving the remaining modified CD11b^+^ cells. We found that, following IR, MC38 tumors in mice transferred with *Bambi*-KD BMCs grew significantly faster, while MC38 tumors in mice transferred with BAMBI-overexpressing BMCs grew remarkably slower compared with those transferred with WT BMCs (Figure 4A). Consistently, we observed a lower level of tumor-infiltrating MDSCs in mice transferred with BAMBI-overexpressing BMCs with IR and higher levels in those transferred with *Bambi*-KD BMCs (Supplemental Figure 6A). These results suggest that increasing BAMBI expression in myeloid cells can improve the response to radiotherapy.

To demonstrate that enhanced efficacy of radiation by BAMBI overexpression can be translated into a clinically relevant strategy, we generated an adeno-associated viral (AAV) vector containing BAMBI driven by a CMV promoter for in situ *Bambi* delivery (hereafter AAV-Bambi). We used an AAV9 capsid, which is efficient and increasingly being utilized in clinical trials (35). AAV-mediated delivery of *Bambi* led to elevated expression of BAMBI in tumor-infiltrated MDSCs, as measured by flow cytometry 24 hours after intratumoral (i.t.) injection (2 × 10^10^ viral particles [v.p.]/dose) (Supplemental Figure 6B). To investigate AAV-Bambi’s antitumor efficacy, we performed in vivo studies in mice bearing MC38 tumors treated with AAV-Bambi (i.t. of 2 × 10^10^ v.p., twice weekly for 2 weeks). Although AAV-Bambi monotherapy did suppress tumor growth, the combination of AAV-Bambi and IR (20 Gy, 1 dose) caused a more pronounced suppressive effect on tumor growth compared with that of mice treated with IR alone, assessed in terms of both tumor size (*P* = 0.0018) and animal survival (*P* = 0.0189) (Figure 4, B and C).

We observed that both tumor-infiltrating total CD8^+^ T cells and cytotoxic CD8^+^ T cells (IFN-γ^+^CD8^+^) were significantly increased in AAV-Bambi+IR–treated mice compared with mice treated with IR alone (Supplemental Figure 6, C and D). To investigate whether MDSCs are involved in the enhanced T cell function, we tested the suppressive function of tumor-infiltrating MDSCs isolated from AAV-Bambi–treated mice and observed an attenuated suppression function, as evidenced by higher T cell IFN-γ production (Supplemental Figure 6E). Furthermore, we measured the antitumor efficacy of AAV-Bambi in *Lyz2*^cre^CSF1R-DTR mice, whose MDSCs were depleted after DT treatment. As expected, neither AAV-Bambi alone nor AAV-Bambi+IR lost the tumor inhibition ability (Figure 4D), indicating that AAV-Bambi impairs MDSC suppressive function and thereby leads to the enhanced antitumor effect.

In the Lewis lung carcinoma (LLC) spontaneous lung metastasis model, a reduced metastatic burden (size of the lung metastasis) was observed in lungs of mice that received AAV-Bambi+IR treatment compared with mice that received IR alone (*P* = 0.0016; Figure 4E). Similarly, we observed primary tumor control in AAV-Bambi+IR–treated mice compared with control (*P* = 0.0193; Supplemental Figure 6F). These data indicate that AAV-Bambi treatment enhanced the efficacy of radiotherapy through an increase in both local and distal metastasis control. We also investigated whether AAV-Bambi could further increase the efficacy of IR and anti–PD-L1 treatment using a B16 melanoma model, which is resistant to immune-checkpoint blockade (ICB) therapy. Compared with IR+anti-PD-L1 treatment, triple-combination treatment with AAV-Bambi, IR, and anti–PD-L1 resulted in significantly slower tumor growth (Figure 4F). The results demonstrate that AAV-Bambi can augment the response to radiotherapy and radioimmunotherapy combinations.

## Discussion

We report that IR decreases BAMBI expression and the decreased BAMBI in turn activates TGF-β signaling in MDSCs, resulting in a positive immune-suppression feedback loop. BAMBI alters MDSC migration and suppressive function in the context of IR. Our study delineates a previously unknown epitranscriptional mechanism of BAMBI regulation whereby YTHDF2 directly binds to and triggers degradation of *Bambi* transcript in MDSCs. AAV delivery of *Bambi* into tumors not only improves local tumor control, but also suppresses distant metastasis. The IR/YTHDF2/BAMBI/TGF-β circuit in MDSCs represents a previously unrecognized mechanism of extrinsic radioresistance.

Through scRNA-Seq and flow cytometry analysis, we are able to describe the distribution of BAMBI among immune cells in cancer patient PMBCs and murine colon (MC38) tumors in the context of IR. Considering that BAMBI is expressed at various levels in immune cells, we speculate that BAMBI may affect their function and thereby affect host tumor immune response. Few studies have focused on the role of BAMBI in immune cells. For instance, the Merino group demonstrated that BAMBI modulates the differentiation of CD4^+^ cells during autoimmune arthritis (36). The functional role of BAMBI needs to be further explored in other immune populations, especially in the setting of cancer.

Our findings that TGF-β signaling is required for MDSC migration and suppressive function after IR support previous observations that myeloid-specific TGF-β signaling is a critical mediator of tumor progression (20, 21). We demonstrate that TGF-β signaling is under control of an YTHDF2/BAMBI axis in MDSCs, providing the solid link with m^6^A modification and TGF-β signaling at the receptor level. We demonstrate that NF-κB/RELA plays a central role in YTHDF2-mediated BAMBI regulation in MDSCs. Of note, we cannot rule out the possibility that other NF-κB subunits might be involved in the regulation of BAMBI. For example, P50 could recruit HDAC1 to suppress *Bambi* transcription in hepatic stellate cells (37). We hypothesize that the IR/NF-κB axis contributes to MDSC migration and suppressive function via distinct mechanisms in the context of distinct conditions, resulting in an intricate network of NF-κB signaling in MDSCs. These insights require detailed future mechanistic investigation.

Enhancing BAMBI in myeloid cells not only boosts the local antitumor effects of radiation, but also suppresses distant metastasis, amplifying the clinical importance of our findings. There are numerous anticancer pharmacological interventions that target specific mediators of the TGF-β signaling pathway or TGF-β activators that have been tested in human clinical trials (38). However, adverse effects have presented a major challenge to the implementation of anti–TGF-β therapies, likely due to the dual nature of TGF-β signaling, which can both function as a tumor suppressor in carcinoma cells and promote tumor progression in other contexts, especially in immune cells (39, 40). We demonstrate that BAMBI is mainly expressed in myeloid cells, especially MDSCs, and therefore may be a better target (elevating myeloid cell–specific BAMBI) for inhibiting TGF-β signaling than the systemic TGF-β blockade. We concede that AAV-Bambi is an experimental delivery strategy that was employed to demonstrate the antitumor efficacy of enhancing BAMBI in murine cancer models. More potent Bambi delivery therapies for clinical translation need to be developed in the future. Despite this, our findings reveal detailed insights into therapeutically targeting BAMBI for the treatment of cancer.

## Methods

### Cells and reagents.

MC38 and B16F1 were purchased from ATCC and were maintained according to the method of characterization used by ATCC. LLC cells were obtained from ATCC (CRL-1642). Depleting or blocking antibodies against PD-L1 were purchased from Bio X Cell (catalog BE0101; clone 10F.9G2). AAV-Bambi was produced by Vector BioLabs (QB59234).

### Mice.

All animals were maintained in pathogen-free conditions. *Ythdf2^fl/fl^* mice were generated using CRISPR/Cas9 technology as described (41). *Lyz2*^Cre^ mice, CD45.1 mice, *Rela^fl/fl^* mice, *Ccr2*^–/–^ mice, and *Tgfbr2^fl/fl^* mice were purchased from The Jackson Laboratory. We used female mice of 6 to 8 weeks of age for all experiments.

### Patient sample.

Patient samples (PBMCs) were obtained from patients treated at the University of Chicago enrolled in trial NCT03223155 (31).

### Tumor growth and treatment.

Either 1 ×10^6^ MC38, LLC, or B16F1 tumor cells were s.c. injected in the right flank of mice. Mice were pooled and randomly divided into different groups when the tumor reached a volume of approximately 100 mm^3^ ([length (L) × width (W) × height (H)] × 0.5), but not blinded. The mice were treated with 20 Gy of tumor-localized radiation (1 dose) or sham treatment. For anti–PD-L1 treatment experiments, 200 μg of the anti–PD-L1 antibody were injected i.p. twice each week for a total of 3 times. For AAV-Bambi delivery experiments, the tumor-bearing mice were injected i.t. with 2 × 10^10^ v.p. of either AAV-Bambi or AAV-null, twice each week for a total of 4 times. For adoptive transfer, 5 × 10^6^ MDSCs were injected i.v. into recipient mice (chimeric mice); on the same day, mice received 20 Gy of tumor-localized radiation (1 dose). Tumors were measured twice a week for 3 to 4 weeks. Animals were euthanized when the tumor volume reached 2,000 mm^3^ or the diameter of the tumor reached 1.5 cm (according to the IACUC protocol).

### Overexpression or KD of BAMBI in tumor cells.

The mouse *Bambi* cDNA was cloned into the lentiviral expression vector pLJM1 to construct the vector pLJM1-BAMBI and packaged by cotransfection of 293T cells with 2 lentiviral helper plasmids, pMD2.G and psPAX2. Virus-containing conditioned medium was harvested 48 hours to 72 hours after transfection, filtered, and used to infect MC38 or B16F1 cells in the presence of 8 μg/mL polybrene. Infected cells were selected with 2 to 10 μg/mL puromycin. We knocked down *Bambi* in both MC38 and B16F1 cells using an shRNA approach. The overexpression or KD of cells was confirmed by Western blot analysis.

### Flow cytometry.

For flow cytometric analysis, tumors were collected from mice. The collected tumor tissues were cut into small pieces and digested with 1 mg/ml collagenase type I (Thermo Fisher Scientific) and 200 μg/ml DNase I (Sigma-Aldrich) at 37°C for 60 minutes to generate single-cell suspensions. Samples were then filtered through a 70 μm cell strainer and washed twice with staining buffer (PBS supplemented with 2% FBS and 0.5 mM EDTA). The cells were resuspended in staining buffer and were blocked with anti-FcR (clone 2.4G2; catalog BP0307; Bio X Cell). Subsequently, cells were stained with approximately 100- to 200-fold diluted fluorescence-labeled antibodies for 30 minutes at 4°C in the dark and then detected by flow cytometry with a BD Fortessa. For BAMBI staining, the primary anti-BAMBI antibody (catalog PA5-38027 or catalog PA5-93037; Thermo Fisher Scientific) was first conjugated with secondary antibody with Alexa Fluor 488 or AF647 Conjugation Kit (Fast) — Lightning-Link (Abcam) and then was used for flow staining. Analysis of flow cytometry data was performed using FlowJo, version 10.

Multicolor spectral flow cytometric analysis was performed on patient PBMCs using a 28-color antibody panel on Cytek Aurora (Cytek Biosciences). 5 × 10^4^ live CD45^+^ cells from each sample were concatenated and used for further downstream analysis. High-dimensional data were visualized using *t*-distributed stochastic neighbor embedding (*t*-SNE) in FlowJo, version 10.8.1 (BD). Phenograph, version 2.4 (42), and FlowSOM, version 3.0.18 (43), were used for unsupervised nearest-neighbor clustering based on phenotypic similarities. FlowJo plugin Cluster explorer, version 1.7.4 (BD), was used for data visualization.

### BM-MDSC induction and isolation.

BM was obtained from WT (CD45.1 and CD45.2), *Ythdf2^fl/fl^*, or *Lyz2*^cre^*Ythdf2^fl/fl^* mice and was used to prepare single-cell suspension (fresh BMCs). The cells were cultured in RPMI-1640 medium containing 10% FBS and 20 ng/ml recombinant mouse GM-CSF (carrier-free) (BioLegend). Fresh medium supplemented with GM-CSF was added on day 3. On day 4, the BM-MDSCs were obtained from fresh BMCs, followed with MDSC isolation using EasySep Monocytes Selection Kits (STEMCELL Technologies).

### MDSC suppression assay.

Murine MDSCs purified from tumors or BM-MDSCs were used for the suppression assay. CD8^+^ T cells were isolated from the spleens of naive mice using the EasySep Mouse CD8^+^ T Cell Isolation Kit (STEMCELL Technologies) according to the manufacturer’s instructions and then stained with CellTrace CFSE (Invitrogen). The CD8^+^ T cells were cultured with anti-CD3/anti-CD28 beads and were cocultured with MDSCs at a ratio of 4:1. CD8^+^ T cell proliferation was analyzed by flow cytometry, or the IFN-γ concentration was measured using the CBA Flex Set (BD).

### KD of Bambi in BM-MDSCs.

siRNA targeting mouse *Bambi* was transfected into BM-MDSCs by TransIT-TKO Transfection Reagent (Mirus) according to the manufacturer’s protocol. The sequence of siRNA was as follows: (sense) 5′-CCAGACUUCUCGAUCCUC-Att-3′. One to two days after transfection, the cells were collected. KD efficiency was detected by qPCR.

### Transwell migration assay.

We used 24-well Transwell plates with 8 μm inserts in polyethylene terephthalate track–etched membranes (Corning). The purified MDSCs from tumors or BM-derived cells (1.5 × /10^6^ cells/insert) in serum-free medium were added into the upper compartment of the chamber. The inserts were placed in plates with DMEM medium with 5% FBS. After incubating overnight, insert membranes were washed with PBS, fixed with 70% methanol for 10 minutes, and stained with 0.05% crystal violet to detect the migrated cells. An inverted microscope was used for counting.

### RNA stability assay.

MDSCs were sorted from spleen in *Ythdf2^fl/fl^* and *Lyz2^cre^Ythdf2^fl/fl^* mice and were seeded in 24-well plates at 50% confluency. For IR treatment, MDSCs were treated with IR (4 Gy) and cultured for 6 hours, and 5 μg/mL of actinomycin D (Sigma-Aldrich) was added. After 0, 0.5, 1, 3, and 6 hours of incubation, cells were collected. The total RNA was purified using the RNeasy kit with an additional DNase-I digestion step on the column. RNA quantities were determined using qPCR.

### Forced expression of m^6^A-binding site–mutated YTHDF2 in MDSCs.

The cloned *Ythdf2* cDNA with K416A, R527A, W432A, and W486A mutations, which has been proved to significantly decrease m^6^A-binding affinity (44), was synthesized and cloned into the lentiviral expression vector pLVX-Zsgreen-N1 to generate pLVX-Zsgreen-N1-Ythdf2-Mut (GenScript). The constructed vectors were packaged by cotransfection of ×293 cells with 2 lentiviral helper plasmids, pVSVG and pVPR. Virus-containing conditioned medium was harvested 48 hours after transfection, filtered, and used to infect BM-MDSCs in the presence of 8 μg/mL polybrene. Infected cells were selected with 2 μg/mL puromycin.

### Generation of BM chimeras.

WT mice were irradiated with a single dose of 8 Gy. The irradiated mice were adoptively transferred (i.v.) with 5 × 10^6^ WT BMCs from CD11b-DTR mice. The mice were treated with Neomycin (0.5 mg/mL) diluted in drinking water for 4 weeks after reconstitution and then were used for the adaptive transfer experiments after 8 weeks.

### RIP-qPCR analysis.

RIP for YTHDF2 was performed using 10 to 20 μg anti-YTHDF2 rabbit polyclonal antibody (catalog ARP67917_P050; Aviva Systems Biology). After IP, RNA was isolated from input and IP fractions using phenol/chloroform extraction. cDNA was prepared with the Applied Biosystems High-Capacity cDNA Reverse Transcription Kit (Thermo Fisher Scientific). SYBR Green–based qPCR was performed using QuantiStudio3 (ABI).

### Immunoblot analysis.

Whole-cell protein was extracted with RIPA buffer supplemented with Protease/Phosphatase Inhibitor Cocktail (×100) (Thermo Scientific). Protein concentrations were measured with the BCA Protein Assay Kit (Pierce). Equal amounts of protein were separated by SDS-PAGE and transferred to PVDF membrane (Invitrogen). Membranes were blocked in 1% BSA or 5% milk for 1 hour and incubated with the relevant primary antibodies at 4°C overnight. Next, the membrane was washed with Tris-buffered saline with 0.1% Tween 20 (TBST) and incubated with the appropriate secondary HRP antibody (catalog sc-2357; Santa Cruz Biotechnology Inc.) for another 1 hour. The membrane was then washed with TBST and ECL was applied for film development. The amount of loaded protein was normalized to β-actin.

### scRNA-Seq analysis.

CD45^+^ single-cell suspensions were obtained from 4 pooled MC38 tumors in WT mice with or without IR (20 Gy) 4 days after IR. Raw scRNA-Seq data were processed into FASTQ files using the cellranger mkfastq function of 10x Genomics Cell Ranger (version 6.0.1), and the reads were then aligned to the mouse reference genome mm10 (45). The unique molecular identifier (UMI) for each gene in each cell was counted with the cellranger count function (45). Then low-quality cells were discarded if they contained (a) fewer than 200 expressed genes or (b) more than 25% mitochondrial genes. The doublets were removed by DoubletFinder (version 2.0.3) assuming 6% doublet formation rate (46). The processed whole gene expression matrix was then fed to Seurat (version 4.0.6) for downstream analysis (47).

Further, the UMI count matrices from control and IR samples were integrated by the Seurat:IntegrateData method after normalization with the SCT method using 3,000 highly variable feature genes (47). Clustering analyses were performed using the first 40 principal components for constructing the shared nearest neighbor (SNN) graph with resolution as 0.7. The marker genes were called by Seurat:FindAllMarkers (pct.1 ≥ 0.6 and Log_2_FC ≥ 1). Then, identified clusters were annotated by scClassify (version 1.2.0) (48) based on cell types of hierarchies constructed from the reference data set (E-MTAB-8832, CD45^+^ immune cells sorted from MC38 tumor–bearing C57BL/6 mice) (49) and labeled using the selected feature gene. The myeloid cells, which were gathered closely and annotated as macrophage, monocyte, or DC2 cells, were extracted and were clustered into several subclusters. They were further annotated using the same methods as above. Heatmap of *Bambi* expression was plot based on the average normed reads count of this gene across cells from IR or control sample.

### AAV-Bambi production.

Mouse BAMBI cDNA open reading frame (GenBank NM_026505.2) was cloned into the pcDNA3.1+/C-(K)-DYK vector and made available by Genscript. The BAMBI-DYK was subsequently subcloned into a pAAV cis-plasmid driven by CMV, with AAV2 ITR to make AAV9 capsid by Vector BioLabs. The titer of the particles was 2.6 × 10^13^ GC/mL.

### Statistics.

To estimate the statistical significance of differences between 2 groups, we used unpaired (or paired) Student’s *t* tests to calculate 2-tailed *P* values. One-way ANOVA or 2-way ANOVA with multiple-comparison test was performed when more than 2 groups were compared. Survival analysis was performed using Kaplan-Meier curves and evaluated with log-rank Mantel-Cox tests. Error bars represent SEM unless otherwise noted. Statistical analyses were performed using GraphPad Prism (version 9.0).

### Study approval.

The COSINR study was approved by the University of Chicago Biological Sciences Division IRB (IRB17-0547). The studies complied with all ethical regulations, and all patients provided written, informed consent. The mouse study was approved by the IACUC of the University of Chicago (animal protocol #70931) and the Institutional Biosafety Committee (#IBC1517). All animals were maintained in pathogen-free conditions and cared for in accordance with the International Association for Assessment and Accreditation of Laboratory Animal Care policies and certification.

### Data availability.

This study analyzed the existing scRNA-Seq, RIP-Seq, and m^6^A-Seq data sets from NCBI’s Gene Expression Omnibus (GEO) accession number GSE206387 (28). Values for all data points in graphs are reported in the Supporting Data Values file. Any additional information required to reanalyze the data reported in this paper is available upon request.

## Author contributions

LW, HLL, and RW designed the study. LW performed most of the experiments. XY, WS, and X Dou performed bioinformatics analysis. AP and HLL performed patient PBMC flow analysis. WS, X Ding, CW, XH, JW, DC, LZ, and KY helped to perform animal experiments and in vitro experiments. AA, HLL, and CH provided scientific guidance for the research. SP and SJC provided clinical trial samples. LW and RW wrote the manuscript. JB, HLL, AP, and CH helped to edit the manuscript. The order of shared first authorship is based on equal contribution and based on temporal sequence of contribution.

## Supplementary Material

Supplemental data

Supporting data values

## Version 1 12/15/2023

Electronic publication
