# Supplementary material for: Epitranscriptional regulation of TGF-β pseudoreceptor BAMBI by m6A/YTHDF2 drives extrinsic radioresistance
Source: J Clin Invest. 2023 Dec 15;133(24):e172919. doi: 10.1172/JCI172919 (PMC10721150; doi:10.1172/JCI172919)
Supplement: Supplemental data [file jci-133-172919-s183.pdf]

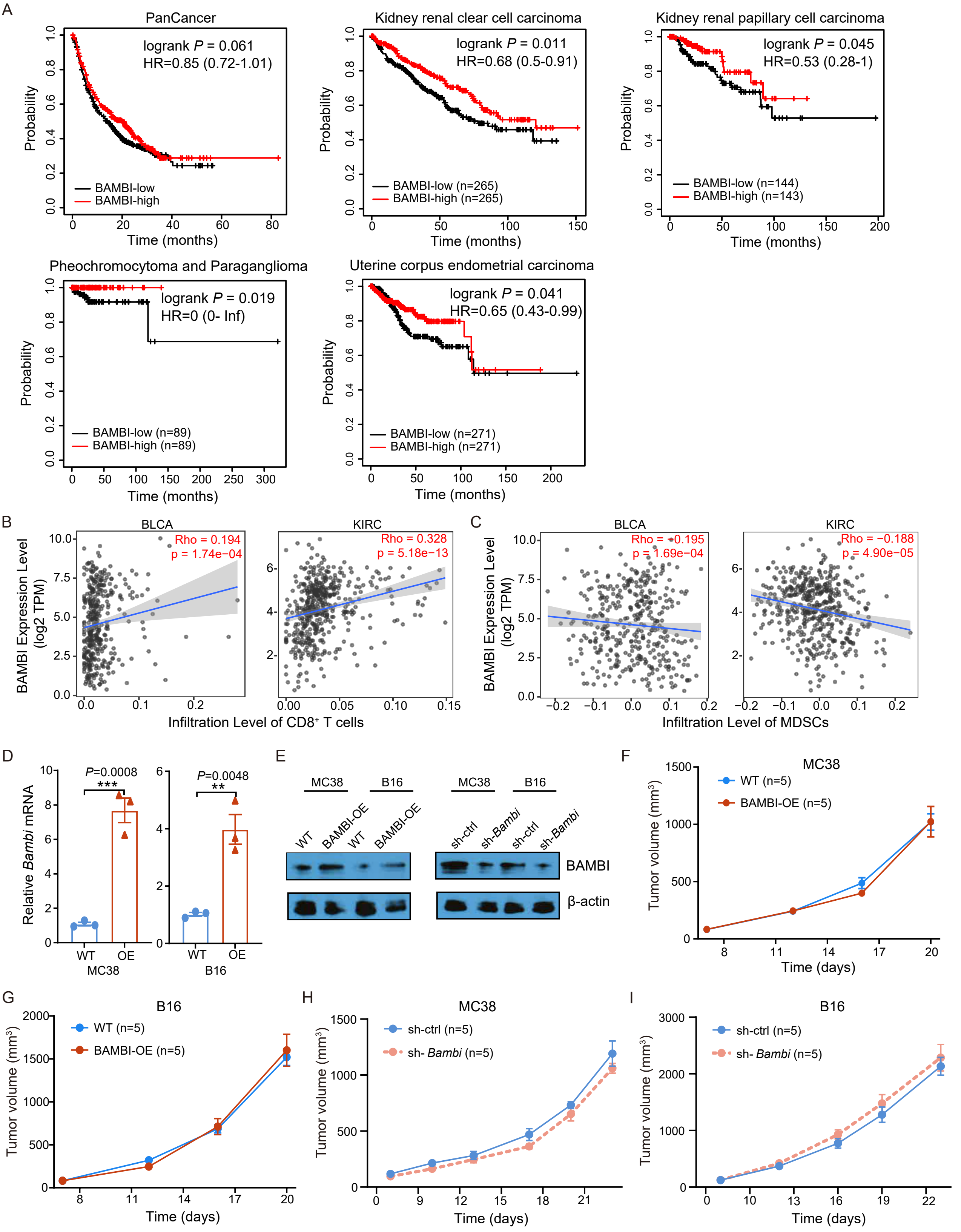

A
PanCancer
logrank P = 0.061
HR=0.85 (0.72-1.01)
BAMBI-low
BAMBI-high
Probability
Time (months)
Kidney renal clear cell carcinoma
logrank P = 0.011
HR=0.68 (0.5-0.91)
BAMBI-low (n=265)
BAMBI-high (n=265)
Kidney renal papillary cell carcinoma
logrank P = 0.045
HR=0.53 (0.28-1)
BAMBI-low (n=144)
BAMBI-high (n=143)
Pheochromocytoma and Paraganglioma
logrank P = 0.019
HR=0 (0- Inf)
BAMBI-low (n=89)
BAMBI-high (n=89)
Uterine corpus endometrial carcinoma
logrank P = 0.041
HR=0.65 (0.43-0.99)
BAMBI-low (n=271)
BAMBI-high (n=271)
B
BLCA
Rho = 0.194
p = 1.74e−04
KIRC
Rho = 0.328
p = 5.18e−13
BAMBI Expression Level (log2 TPM)
Infiltration Level of CD8⁺ T cells
C
BLCA
Rho = −0.195
p = 1.69e−04
KIRC
Rho = −0.188
p = 4.90e−05
BAMBI Expression Level (log2 TPM)
Infiltration Level of MDSCs
D
Relative Bambi mRNA
P=0.0008
***
P=0.0048
**
WT
OE
MC38
B16
E
MC38
B16
WT
BAMBI-OE
sh-ctrl
sh-Bambi
BAMBI
β-actin
F
MC38
WT (n=5)
BAMBI-OE (n=5)
Tumor volume (mm³)
Time (days)
G
B16
WT (n=5)
BAMBI-OE (n=5)
H
MC38
sh-ctrl (n=5)
sh- Bambi (n=5)
I
B16
sh-ctrl (n=5)
sh- Bambi (n=5)

Supplemental Figure S1. BAMBI expression correlates with patient's outcome and immune responses.

(A) Overall survival analysis of cancer patients in PanCancer, kidney renal clear cell carcinoma (KIRC), kidney renal papillary cell carcinoma (KIRP), pheochromocytoma and paraganglioma (PCPG), and uterine corpus endometrial carcinoma cohorts (UCEC) using the median value as a cutoff. High-BAMBI (expression ≥ median value); Low-BAMBI (expression < median value). Normalized gene expression and corresponding clinical data on patients were obtained from KM-plotter.

(B-C) Correlation of BAMBI expression and infiltration level of $CD8^+$ T cells (**B**) and MDSCs (**C**) in Bladder Carcinoma (BLCA) and KIRC cohorts, obtained by TIMER2.0. R represents Spearman's coefficient.

(D) Relative mRNA level of *Bambi* in WT and BAMBI-overexpressing (BAMBI-OE) MC38 (left) and B16F1 (right) cells. (n= 3 per group)

(E) Immunoblot analysis of BAMBI in WT and BAMBI-OE MC38 or BAMBI-OE B16F1 cells (Left). Immunoblot analysis of BAMBI in WT (sh-ctrl) and BAMBI-KD (sh-*Bambi*) MC38 or B16F1 cells (Right).

(F-G) WT C57BL/6 mice were injected subcutaneously with 1×10^6 MC38 or BAMBI-OE MC38 cells (**F**) and B16F1 or BAMBI-OE B16F1 cells (**G**). Tumor growth was monitored. (n= 5 mice per group)

(H-I) WT C57BL/6 mice were injected subcutaneously with 1×10^6 MC38 (sh-ctrl) or BAMBI-KD (sh-*Bambi*) MC38 cells (**H**) and B16F1 (sh-ctrl) or BAMBI-KD (sh-*Bambi*) B16F1 cells (**I**). Tumor growth was monitored. (n= 5 mice per group)

Data are represented as mean ± s.e.m. One of two or three representative experiments was shown (D-I). Statistical analysis was performed using two-sided unpaired Student's *t*-test (D) or two-way ANOVA test with corrections for multiple variables (F-I). $^*P < 0.05$, $^{**}P < 0.01$, and $^{***}P < 0.001$.

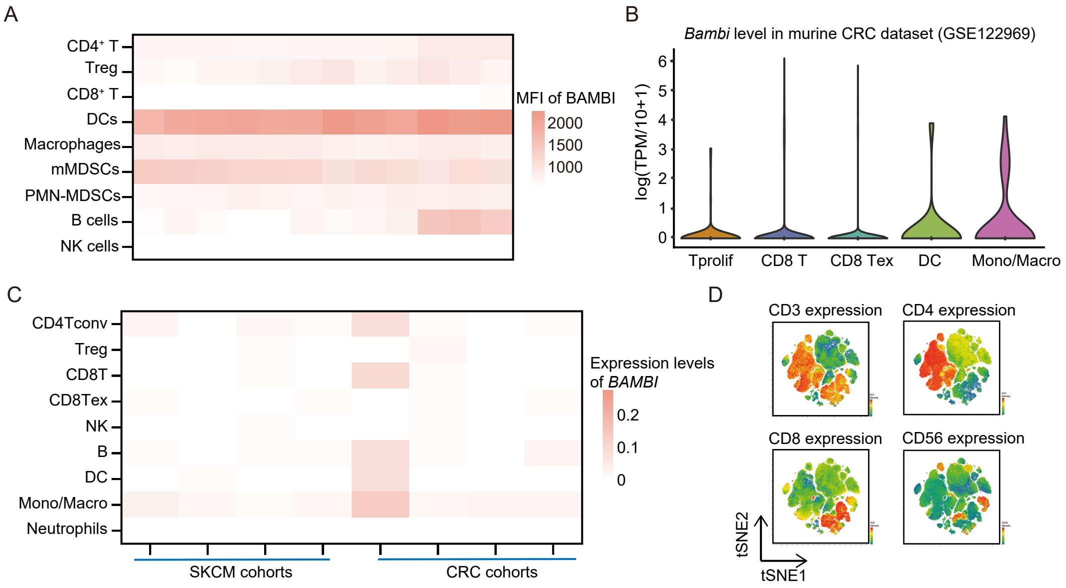
A
CD4⁺ T
Treg
CD8⁺ T
DCs
Macrophages
mMDSCs
PMN-MDSCs
B cells
NK cells
MFI of BAMBI
2000
1500
1000
B
Bambi level in murine CRC dataset (GSE122969)
log(TPM/10+1)
0
1
2
3
4
5
6
Tprolif
CD8 T
CD8 Tex
DC
Mono/Macro
C
CD4Tconv
Treg
CD8T
CD8Tex
NK
B
DC
Mono/Macro
Neutrophils
Expression levels of *BAMBI*
0.2
0.1
0
SKCM cohorts
CRC cohorts
D
CD3 expression
CD4 expression
CD8 expression
CD56 expression
tSNE2
tSNE1

Supplemental Figure S2. Distribution of BAMBI expression in different immune cells in both humans and murine cancers.

(A) Heatmap showing the Mean Fluorescent Intensity (MFI) of BAMBI in different MC38 tumor-infiltrating immune cells, including $CD4^+$ T cells ($CD45^+CD4^+$), Treg cells ($CD45^+CD4^+CD25^+$), $CD8^+$ T cells ($CD45^+CD8^+$), DCs ($CD45^+CD11c^+MHCII^+$), Macrophages ($CD45^+CD11b^+F4/80^+$), mMDSCs ($CD45^+CD11b^+Ly6C^{hi}Ly6G^-$), PMN-MDSCs ($CD45^+CD11b^+Ly6C^-Ly6G^+$), B cells ($CD45^+CD3^-CD19^+$), and NK cells ($CD45^+CD3^-NK1.1^+$).

(B) Violin plot showing the mRNA levels of *Bambi* in different immune cells based on the public scRNA-seq data of murine colon cancer (GSE122969), obtained by TISCH2.

(C) Heatmap showing the mRNA levels of *BAMBI* in different immune cells based on the public scRNA-seq data of SKCM and CRC patients, obtained by TISCH2.

(D) tSNE clustering of flow cytometry marker expression profiles in live $CD45^+$ cells of PBMCs from metastatic NSCLC patients enrolled in a clinical trial (the COSINR study, NCT03223155). Expression intensity of CD3, CD4, CD8 and CD56.

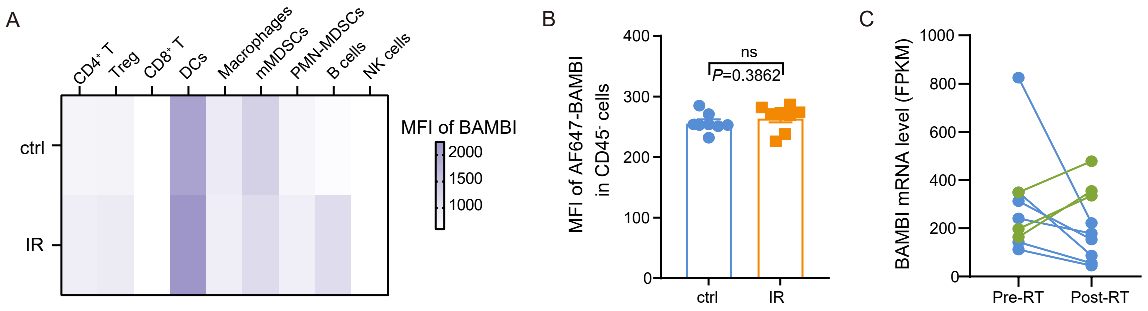
A
CD4+ T
Treg
CD8+ T
DCs
Macrophages
mMDSCs
PMN-MDSCs
B cells
NK cells
ctrl
IR
MFI of BAMBI
2000
1500
1000
B
MFI of AF647-BAMBI in CD45- cells
400
300
200
100
0
ns
P=0.3862
ctrl
IR
C
BAMBI mRNA level (FPKM)
1000
800
600
400
200
0
Pre-RT
Post-RT

Supplemental Figure S3. IR specifically reduces BAMBI expression in MDSCs.

(A) Heatmap showing the Mean Fluorescent Intensity (MFI) of BAMBI in different MC38 tumor-infiltrating immune cells, (ctrl *vs.* IR, three days after IR) including $CD4^+$ T cells ($CD45^+CD4^+$), Treg cells ($CD45^+CD4^+$ $CD25^+$), $CD8^+$ T cells ($CD45^+CD8^+$), DCs ($CD45^+CD11c^+MHCII^+$), Macrophages ($CD45^+CD11b^+F4/80^+$), mMDSCs ($CD45^+CD11b^+Ly6C^{hi}Ly6G^-$), PMN-MDSCs ($CD45^+CD11b^+Ly6C^-Ly6G^+$), B cells ($CD45^+CD3^-$ $CD19^+$), and NK cells ($CD45^+CD3^-NK1.1^+$).

(B) Mean Fluorescent Intensity (MFI) of BAMBI in $CD45^-$ cells in nonirradiated (ctrl) *vs.* irradiated (IR) MC38 tumors. (n=8 per group)

(C) The *BAMBI* mRNA levels in different cancer patients with lung or liver metastasis, based on the RNA-seq of whole tumor biopsy (pre-RT *vs.* post-RT, the COSINR study, NCT03223155).

Data are represented as mean ± s.e.m. One of two representative experiments was shown (B). Statistical analysis was performed using two-sided unpaired Student's *t*-test (B) or two-sided paired Student's *t*-test (C).

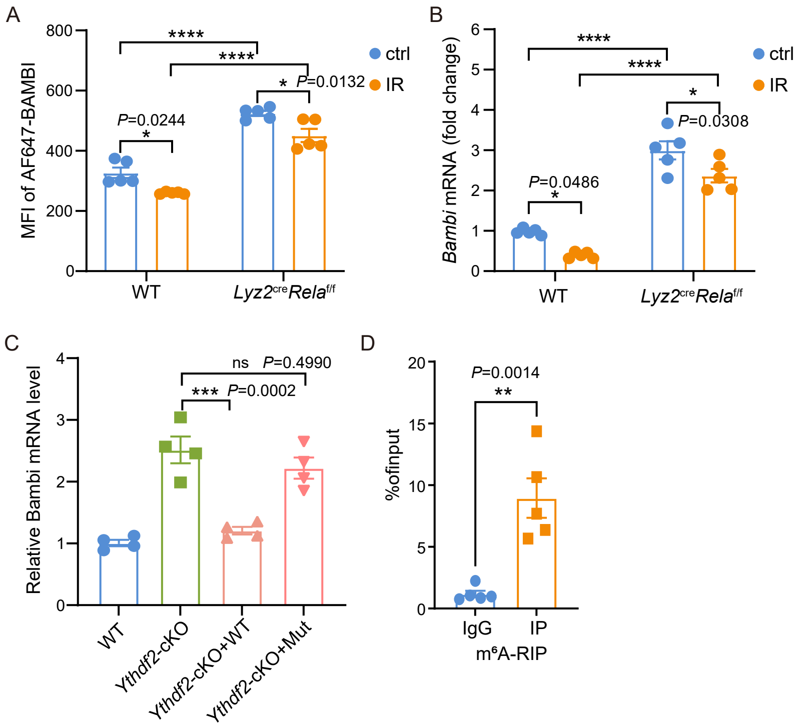
A
MFI of AF647-BAMBI
800
600
400
200
0
****
****
* P=0.0132
P=0.0244
*
ctrl
IR
WT
Lyz2creRelaf/f
B
Bambi mRNA (fold change)
6
5
4
3
2
1
0
****
****
*
P=0.0308
P=0.0486
*
ctrl
IR
WT
Lyz2creRelaf/f
C
Relative Bambi mRNA level
4
3
2
1
0
ns P=0.4990
*** P=0.0002
WT
Ythdf2-cKO
Ythdf2-cKO+WT
Ythdf2-cKO+Mut
D
%ofinput
20
15
10
5
0
P=0.0014
**
IgG
IP
m6A-RIP

Supplemental Figure S4. YTHDF2 down-regulates BAMBI expression in MDCSs.

(A) Mean Fluorescent Intensity (MFI) of BAMBI in MDSCs in nonirradiated (ctrl) vs. irradiated (IR) MC38 tumors (three days after IR) from WT mice and *Lyz2*^{cre}*Rela*$^{f/f}$ mice. (n= 5 per group)

(B) qPCR analysis of *Bambi* in MDSCs (CD45^{+}CD11b^{+}Ly6C^{hi}) isolated from nonirradiated (ctrl) *vs*. irradiated (IR, one dose of 20Gy) MC38 tumors (three days after IR) from WT mice and *Lyz2*^{cre}*Rela*$^{f/f}$ mice. (n= 5 per group)

(C) The WT-YTHDF2 and m^{6}A-binding-site-mutated YTHDF2-overexpressing *Ythdf2*-deficient CD45.2-BM-MDSCs (*Ythdf2*-cKO+WT, *Ythdf2*-cKO+Mut respectively) were obtained via lentivirus transfection. The BM-MDSCs were used for adoptive transfer into MC38 tumor-bearing CD45.1 mice. On the same day, the mice were treated with tumor local irradiation (one dose of 20 Gy). Three days after IR, tumors were harvested to measure the mRNA level of *Bambi* (by qPCR analysis) in newly infiltrated CD45.2-MDSCs by flow cytometry. (n= 4 per group)

(D) The Graphs showing enrichment of *Bambi* mRNA in the m^{6}A-immunoprecipitated RNA fraction of BM-MDSCs, determined by RIP-qPCR. Rabbit IgG served as a control. Enrichment of the indicated genes was normalized to the input level. (n = 5 per group).

Data are represented as mean ± s.e.m. One of two or three representative experiments was shown. Statistical analysis was performed using one-way ANOVA with Bonferroni's multiple comparison tests (A-C) or two-sided unpaired Student's *t*-test (D). *$P < 0.05$, **$P < 0.01$, ***$P < 0.001$ and ****$P < 0.0001$.

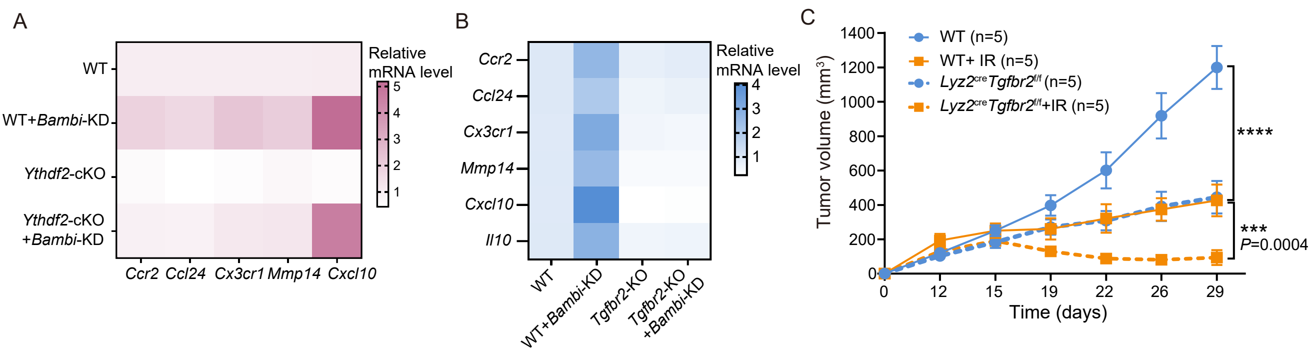

A
WT
WT+Bambi-KD
Ythdf2-cKO
Ythdf2-cKO +Bambi-KD
Ccr2 Ccl24 Cx3cr1 Mmp14 Cxcl10
Relative mRNA level
5
4
3
2
1
B
Ccr2
Ccl24
Cx3cr1
Mmp14
Cxcl10
Il10
WT
WT+Bambi-KD
Tgfbr2-KO
Tgfbr2-KO +Bambi-KD
Relative mRNA level
4
3
2
1
C
WT (n=5)
WT+ IR (n=5)
Lyz2creTgfbr2f/f (n=5)
Lyz2creTgfbr2f/f+IR (n=5)
Tumor volume (mm3)
1400
1200
1000
800
600
400
200
0
0
12
15
19
22
26
29
Time (days)
****
***
P=0.0004

Supplemental Figure S5. TGF-β signaling in myeloid cells alters the response to radiotherapy.

(A) Heatmap showing the qPCR analysis of relative *Ccr2*, *Ccl24*, *Cx3cr1*, *Mmp14*, and *Cxcl10* mRNA expression in WT, *Bambi*-KD, *Ythdf2*-cKO (*Lyz2*^{cre}*Ythdf2*$^{f/f}$), and *Bambi*-KD-*Ythdf2*-cKO BM-MDSCs stimulated with murine rTGF-β.

(B) Heatmap showing the qPCR analysis of relative *Ccr2*, *Ccl24*, *Cx3cr1*, *Mmp14*, and *Cxcl10* mRNA expression in WT, *Bambi*-KD, *Tgfbr2*-KO, and *Bambi*-KD-*Tgfbr2*-KO BM-MDSCs stimulated with murine rTGF-β.

(C) WT (*Tgfbr2*$^{f/f}$) or *Lyz2*^{cre}*Tgfbr2*$^{f/f}$ mice were injected subcutaneously with 1 x 10^6 MC38 cells. When the tumor size reached 100 mm^3, tumor-bearing mice were treated with tumor-local IR (20 Gy, one dose). Tumor growth was monitored. (n= 5 mice per group)

Data are represented as mean ± s.e.m. One of two representative experiments was shown (C). Statistical analysis was performed using two-way ANOVA test with corrections for multiple variables (C). ***$P < 0.001$ and ****$P < 0.0001$.

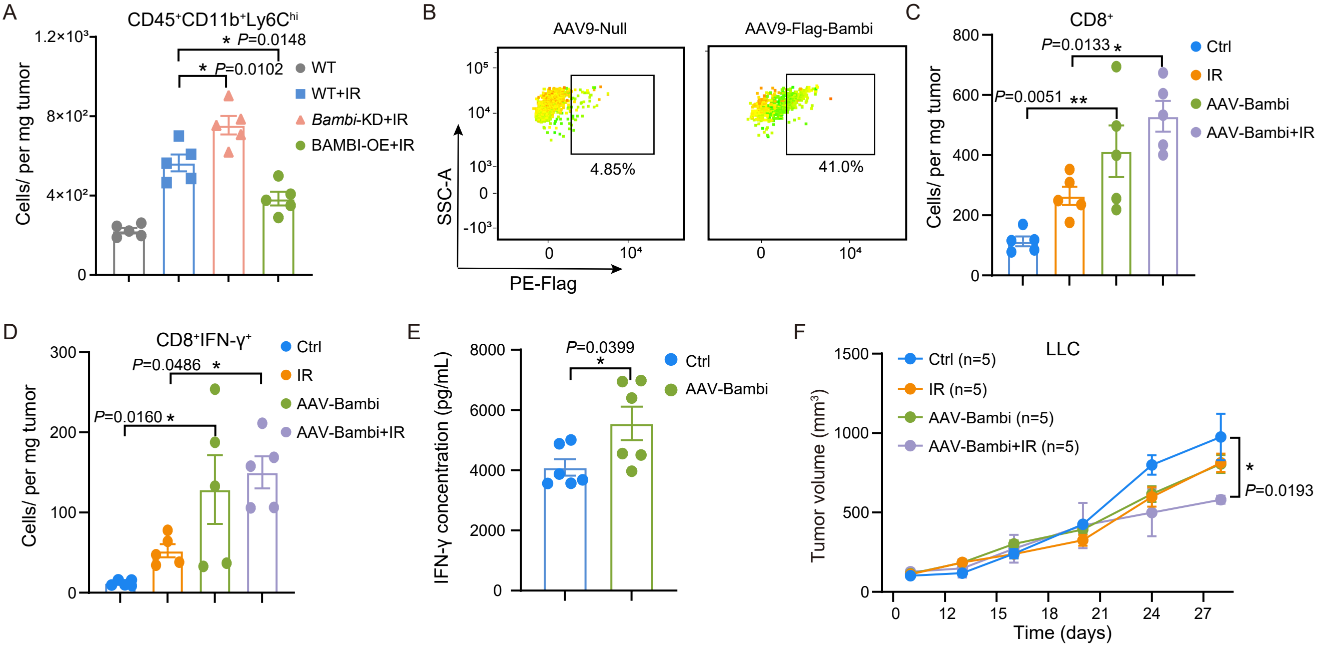
A
CD45+CD11b+Ly6Chi
Cells/ per mg tumor
* P=0.0148
* P=0.0102
WT
WT+IR
Bambi-KD+IR
BAMBI-OE+IR
B
AAV9-Null
AAV9-Flag-Bambi
4.85%
41.0%
SSC-A
PE-Flag
C
CD8+
P=0.0133 *
P=0.0051 **
Ctrl
IR
AAV-Bambi
AAV-Bambi+IR
D
CD8+IFN-γ+
P=0.0486 *
P=0.0160 *
E
IFN-γ concentration (pg/mL)
P=0.0399
F
LLC
Ctrl (n=5)
IR (n=5)
AAV-Bambi (n=5)
AAV-Bambi+IR (n=5)
Tumor volume (mm3)
Time (days)
P=0.0193

Supplemental Figure S6. Combination AAV-Bambi and IR treatment enhances immune responses.

(A) The number of tumor-infiltrating $CD45^+CD11b^+Ly6C^{hi}$ cells in mice transferred with indicated MDSCs, three days after IR, as assessed by flow cytometry. (n= 5 per group)

(B) Representative flow cytometry analysis of PE-(flag) BAMBI levels in tumor-infiltrating MDSCs ($CD45^+CD11b^+Ly6C^{hi}Ly6G^-$), 24h after AAV-Bambi (AAV9-CMV-Flag-Bambi) treatment (i.t. of 2 x 10^{10} v.p.).

(C-D) The numbers of tumor-infiltrating $CD45^+CD8^+$ (**C**) and $CD45^+CD8^+IFN\gamma^+$ (**D**) T cells in MC38 tumor-bearing mice with treatments as indicated seven days after IR. (n = 5 per group)

(E) MDSCs were isolated from MC38 tumors with different treatment as indicated and cocultured with naïve $CD8^+$ T cells for three days. The concentration of IFN-γ was measured by CBA flex set. (n = 6 per group)

(F) LLC tumor-bearing mice were treated with tumor-local IR (20 Gy, one dose). On the same day, the mice were intratumorally injected with 2 x 10^{10} v.p. of AAV-Null (ctrl), AAV-Bambi (twice weekly, three doses totally). The primary tumor growth was monitored. (n = 5 mice per group)

Data are represented as mean ± s.e.m. One of two or three representative experiments was shown. Statistical analysis was performed using one-way ANOVA with Bonferroni's multiple comparison tests (A, C, D), two-sided unpaired Student's *t*-test (E), or two-way ANOVA test with corrections for multiple variables (F). $^*P < 0.05$, and $^{**}P < 0.01$.
